# Determinants of Oral Health: Does Oral Health Literacy Matter?

**DOI:** 10.1155/2013/249591

**Published:** 2013-03-13

**Authors:** Mohammad Mehdi Naghibi Sistani, Reza Yazdani, Jorma Virtanen, Afsaneh Pakdaman, Heikki Murtomaa

**Affiliations:** ^1^Department of Community Oral Health, School of Dentistry, Tehran University of Medical Sciences, P.O. Box 1439955991, Tehran, Iran; ^2^Department of Oral Public Health, Institute of Dentistry, University of Helsinki, P.O. Box 41, 00014 Helsinki, Finland; ^3^Department of Public Health, University of Helsinki, P.O. Box 41, 00014 Helsinki, Finland; ^4^Department of Community Dentistry, University of Oulu, P.O. Box 5281, 90014 Oulu, Finland

## Abstract

*Objective*. To evaluate oral health literacy, independent of other oral health determinants, as a risk indicator for self-reported oral health. *Methods*. A cross-sectional population-based survey conducted in Tehran, Iran. Multiple logistic regression analysis served to estimate the predictive effect of oral health literacy on self-reported oral health status (good versus poor) controlling for socioeconomic and demographic factors and tooth-brushing behavior. *Results*. In all, among 1031 participants (mean age 36.3 (SD 12.9); 51% female), women reported brushing their teeth more frequently (*P* < 0.001) and scored higher for oral health literacy (mean 10.9 versus 10.2, *P* < 0.001). In the adjusted model, high age (OR = 1.01, 95% CI 1.003–1.034), low education (OR = 1.88, 95% CI 1.23–2.87), small living area in square meters per person (OR = 1.85, 95% CI 1.003–3.423), poor tooth brushing behavior (OR = 3.35, 95% CI 2.02–5.57), and low oral health literacy scores (OR = 1.58, 95% CI 1.02–2.45) were significant risk indicators for poor self-reported oral health. *Conclusions*. Low oral health literacy level, independent of education and other socioeconomic determinants, was a predictor for poor self-reported oral health and should be considered a vital determinant of oral health in countries with developing health care systems.

## 1. Introduction

Discrepancies in oral health status result from numerous obstacles ranging from social [1], environmental [2], biological, behavioural [3], cultural, economic, and political factors [3, 4], to limited access to oral health care services, complicated oral health care systems, a lack of oral-health-information material [5], and oral heath literacy [6]. 

The process of acquiring oral health information, appraising its concepts, and applying oral health prevention and treatment plans appropriately requires new skill development called oral health literacy (OHL) [6]. Oral health literacy is an interplay between culture and society, the health system, education system, and oral health outcomes [5, 7] indicating that it may be a new determinant of oral health and should be considered more intensively in oral health research.

Although current research reveals that oral health literacy is associated with the level of education [8, 9], ethnic group [9, 10], dental service utilization, oral health knowledge, and oral self-care behaviour [11], but knowledge about the impact of oral health literacy on oral health outcomes is scarce [12, 13]; moreover, little is known about this association in countries with developing health care systems such as Iran. A poor level of oral hygiene and poor oral health status are evident, especially among lower Iranian socioeconomic groups [14–16].

This study therefore aimed to evaluate the role of oral health literacy as a determinant of oral health among adults in Tehran, Iran. We hypothesized that low oral health literacy level, independent of oral health socioeconomic determinants, is a risk factor for poor self-reported oral health.

## 2. Materials and Methods 

### 2.1. Design and Data Collection

This cross-sectional population-based survey was conducted in 2011 among adults in Tehran, Iran. The Iranian Students' Polling Agency (ISPA), a professional agency, assisted in data collection. All interviewers were trained in a three-hour practical session by the main researcher in order to insure uniformity in data collection and avoid inter-interviewer variability. Training issues were related to the questions and responses, ethical considerations, and way to complete the interview-administered section of the questionnaire. Participants were interviewed at their homes. 

### 2.2. Sampling

A stratified multistage random area served for the sampling frame. The 22 districts of Tehran were considered as strata. The samples were weighted in each stratum based on the proportion of the district population to that of the Tehran population. Within each stratum, regions were selected randomly as clusters, and then blocks were selected from each region randomly. Then ten houses in each block were selected systematically. In the final selection individuals were selected at random among all adults residing in the same house. Those unable to read or write Persian (local language) were excluded.

A sample size of 1031 was estimated by use of a sample-size calculation for estimating a single proportion (*α* = 0.05, the prevalence of adequate oral health literacy or *P* = 0.4 obtained from pilot study [17], and design effect = 2). 

### 2.3. Measures

The following oral-health-related measures required responses: self-reported oral health, measured by asking each individual a question: “In general, how would you describe your oral health at present?” Five response categories were “excellent,” “very good,” “good,” “fair,” and “poor”. For analysis we combined the categories “excellent,” “very good,” and “good” to yield a measure of “good self-reported oral health” and the categories “fair” and “poor” to yield a measure of “poor.”

*Brushing Behaviour*. We asked each individual to respond to a 4-option statement indicating their tooth-brushing behaviour (rarely or never, 2 to 3 times per week, once daily, twice daily or more). For analysis, we reclassified the brushing behaviour to “less than once daily” (combining the categories of “rarely or never” and “2 to 3 times per week”), “once daily,” and “twice daily or more.” 

*Oral Health Literacy*. We used the newly constructed Oral Health Literacy Adults Questionnaire (OHL-AQ) which was tested in a pilot study and showed that it is a reliable and valid questionnaire [17]. The OHL-AQ comprises four sections: reading comprehension, numeracy, listening, and decision-making. 

The reading comprehension section consisted of six items with words omitted from one passage (3 sentences) on oral health knowledge. Of the four possible choices for each omitted word, one was correct and one choice (do not know) was added to avoid guessing or avoid missing responses. For instance, in the sentence “Brushing with toothpaste that contains [___] at least twice a [___]…… could prevent tooth decay,” four options for the first omitted word were flavors, whitening agent, detergent, and fluoride, and for the second month, meal, day, and week. This section was self-administered, with all respondents instructed to read the paragraph and fill in the blanks [17].

**Figure 1:**
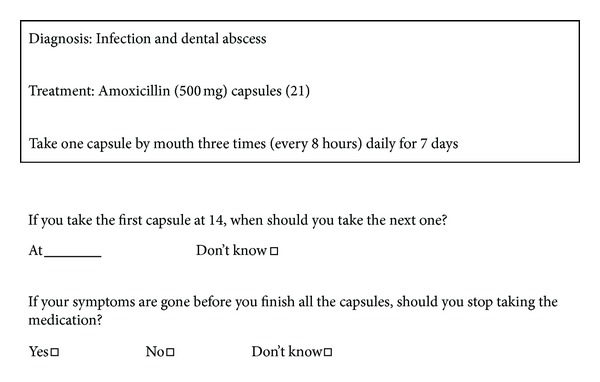
Oral health literacy adults questionnaire (OHL-AQ), amoxicillin consumption prescription.

The numeracy section consisted of four questions related to two topics: an amoxicillin consumption prescription (2 questions) modified from the OHLI [18] and instructions for a sodium fluoride mouth rinse (2 questions). The prescription and instructions were added in a written box, and participants were instructed to write or select the answers (see Figure 1).

The listening section consisted of two questions about instructions after tooth extraction. This was interviewer-administered section. The interviewer read three sentences of postextraction instruction aloud twice, while participants listened and then wrote the answers. The decision-making section contained five questions related to common oral health problems and items extracted from a medical history form. Participants were instructed to read the questions and select one of four possible choices [17]. 

The interviewers did not help participants in reading, answering, or in conceptual meaning of items. The interviewers checked for missing items and asked the participants to answer those or select the “do not know” alternative. The correct answers were scored 1; the incorrect, do not know, and unanswered were scored 0. Then, the sum of the correct answers was calculated to provide the total score for the questionnaire, ranging from 0 to 17. 

Among background information acquired was demographic data comprising age, gender, and socioeconomic measures. We used years of formal education as the measure of social level. Formal education is a valid and reliable indicator for studies of association between health and social status in Iran [19, 20]. Education was categorized into two levels: first level (1–11 years) and second (12 years and more). Economic status was assessed by living area in square meters per person (m^2^/p) [20]. This proxy measure was categorized into three levels: less than 20, 20 to 39, and equal to or more than 40 square meters per person living area.

### 2.4. Statistical Analysis

In addition to descriptive statistics, potential variation in self-reported oral health as an outcome variable was estimated by odds ratios, using multiple logistic regression while adjusting for age, gender, educational level, living area in square meters per person (m^2^/p), tooth-brushing behavior, and oral health literacy as independent variables. We compared good self-reported oral health versus poor self-reported oral health. The significance was set at <0.05. All data were analysed by SPSS software for Windows (version 18).

### 2.5. Ethics

The ethics committee of the Tehran University of Medical Sciences approved the study. Participants were also informed before being invited to this survey about the scientific goal of this research, voluntary participation, and their right to withdraw at any time.

## 3. Results

**Figure 2:**
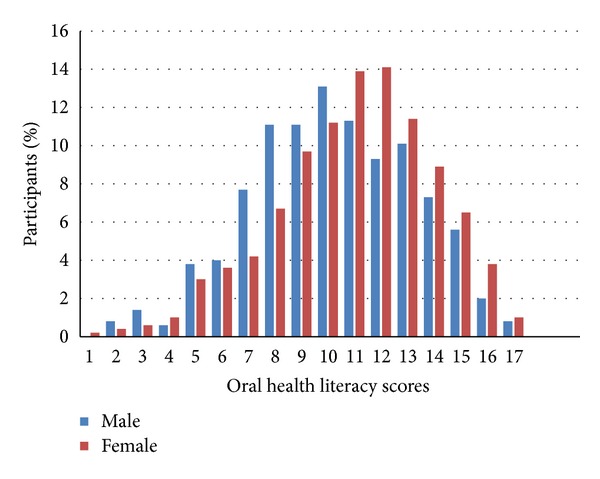
Distribution of oral health literacy adults questionnaire (OHL-AQ) scores by gender among adults in Tehran, Iran (*n* = 1031).

Totally 1031 individuals participated. Characteristics of the study sample are in Table 1. Mean age of participants was 36.3 (SD 12.9) and ranged from 18 to 65 years. Of the whole, 51.1% were female. Women reported brushing their teeth more frequently than men did (*P* < 0.001) and scored higher on the oral health literacy (mean OHL-AQ 10.9 versus 10.2, *P* < 0.001) as well. Oral health literacy scores approximated a normal distribution, 11 being the mode (Figure 2).

The results from multiple logistic regression analysis, both univariate and adjusted model, are in Table 2. High age (OR = 1.01, 95% CI 1.00–1.03), low education (OR lowest level versus upper level = 1.88, 95% CI 1.23–2.87), small living area in square meters per person (OR lowest level versus upper level = 1.85, 95% CI 1.00–3.42), poor tooth-brushing behavior (OR lowest level versus upper level = 3.35, 95% CI 2.02–5.57), and low oral health literacy scores (OR lowest level versus upper level = 1.58, 95% CI 1.02–2.45) were the most significant contributing factors to poor self-reported oral health. 

## 4. Discussion

Among adults queried in Tehran, Iran, it was interesting to note that low oral health literacy level, independent of education and other socioeconomic determinants, was a predictor for poor self-reported oral health. 

Although the association between oral health literacy scores and self-reported oral health was confounded by other variables in the adjusted model, it reached a statistically significant level (OR = 1.58, 95% CI 1.02–2.45); this would confirm our hypothesis that low oral health literacy level contributes to the poor self-reported oral health. Downstream outcomes like oral health status are also affected by numerous determinants other than literacy [8] such as age, education, and economic status. Our findings are in line with earlier ones which have shown that high age [21, 22] and low level of education [23, 24] are related to poor self-reported oral health. 

Since income information in Iran is unreliable and Iranians usually hold more than one job at a time, we used “living area in square meters per person” as a measure of economic status. Similarly one study from Iran [20] revealed “living area in square meters per person” as demonstrating a strong correlation with mortality caused by myocardial infarction. We found that economic status was associated with self-reported oral health in the adjusted model. People who were better off rated their oral health status as better than those with suboptimal living conditions did. This finding is consistent with findings revealing socioeconomic inequalities in relation to oral health status [25–27]. 

Present study showed a significant association between poor self-reported oral health and lack of tooth-brushing (OR lowest level versus upper level = 3.35, 95% CI 2.02–5.57). This would advocate daily tooth-brushing as an inexpensive and easy practice at individual or population level, in order to promote oral health.

Performing population studies on oral health in Iran presents several challenges. Tehran with its 8-million population has become a multicultural metropolitan area with a mixture of socioeconomic and ethnic backgrounds. A sample from Tehran (the capital), however, can at least be considered representative of the urban population of Iran [28]. Access to the national health record data was impossible, and no precise data-recording exists. In order to minimize selection bias, we decided to choose a stratified multistage random area sampling and collect the data at participants' homes. This helped us to increase the response rate as well. 

Social desirability in response to the self-assessment questions could cause response bias [29]. To this reason, the present results should be interpreted cautiously. They could be rather an overestimation of a participant's self-reported oral health status and oral health behavior. Some have found that self-reported oral health measures, however, can serve as a valuable tool in epidemiological studies by reducing resources and costs [30, 31]. This measure has proven useful in evaluating dental conditions and periodontal diseases [32] or in detecting students with healthy oral status; it is more specific than sensitive [33]. 

## 5. Conclusions

Findings from this study indicate that poor oral health literacy was a significant risk indicator for poor self-reported oral health. It seems that oral health literacy should thus deserve recognition as an important determinant of oral health. Indeed, assessment of oral health literacy warrants attention as a priority in oral health promotion programs in countries with developing health care systems.

## Figures and Tables

**Table 1:** Characteristics of the study subjects and descriptive findings by gender.

	Total *N* (%)	Male *N* (%)	Female *N* (%)	P*
Age	1030	504	526	0.89
18–24	251 (24.4)	126 (25.0)	125 (23.8)	
25–44	485 (47.1)	236 (46.8)	249 (47.3)	
45–65	294 (28.5)	142 (28.2)	152 (28.9)	
Mean (SD)	36.3 (12.9)	36.3 (13.2)	36.4 (12.5)	
Years of education	1022	500	522	0.32
<12	280 (27.4)	130 (26.0)	150 (28.7)	
≥12	742 (72.6)	370 (74.0)	372 (71.3)	
Mean (SD)	11.9 (3.8)	12.1 (3.7)	11.6 (3.9)	
Living area (m^2^/p)	947	460	487	0.80
<20	296 (31.3)	148 (32.2)	148 (30.4)	
20–39	485 (51.2)	234 (51.9)	251 (51.5)	
≥40	166 (17.5)	78 (17.0)	88 (18.1)	
Mean (SD)	28.3 (22.0)	28.5 (25.5)	28.1 (18.0)	
Self-reported oral health status	1013	498	515	0.17
Good	856 (84.5)	413 (82.9)	443 (86.0)	
Poor	157 (15.5)	85 (17.1)	72 (14.0)	
Tooth-brushing behaviour	1029	504	525	<0.001
Twice daily or more	387 (37.6)	152 (30.2)	235 (44.8)	
Once daily	450 (43.7)	223 (44.2)	227 (43.2)	
Less than once daily	192 (18.7)	129 (25.6)	63 (12.0)	
Oral health literacy scores	1030	504	526	<0.001
0–9	358 (34.8)	204 (40.5)	154 (29.3)	
10-11	255 (24.8)	123 (24.4)	132 (25.1)	
12–17	417 (40.5)	177 (35.1)	240 (45.5)	
Mean (SD)	10.5 (3.0)	10.2 (3.0)	10.9 (2.9)	

*By Chi-square test.

**Table 2:** Determinants for poor self-assessed oral health based on multiple logistic regression analysis among adults in Tehran, Iran (*n* = 1014).

	OR (95% CI)*	*P*	OR (95% CI)**	*P*
Age (years)	1.02 (1.01–1.03)	<0.001	1.01 (1.00–1.03)	0.02
Gender				
Male	1.00 (ref.)		1.00 (ref.)	
Female	0.79 (0.56–1.11)	0.17	0.96 (0.65–1.40)	0.83
Years of education				
≥12	1.00 (ref.)		1.00 (ref.)	
<12	2.76 (1.94–3.94)	<0.001	1.88 (1.23–2.87)	0.004
Living area (m^2^/p)				
≥40	1.00 (ref.)		1.00 (ref.)	
20–39	1.51 (0.86–2.65)	0.15	1.57 (0.87–2.83)	0.12
<20	2.24 (1.25–3.99)	0.006	1.85 (1.00–3.42)	0.04
Oral health literacy scores				
12–17	1.00 (ref.)		1.00 (ref.)	
10-11	1.41 (0.85–2.25)	0.13	1.08 (0.65–1.80)	0.75
0–9	2.08 (1.40–3.11)	<0.001	1.58 (1.02–2.45)	0.04
Tooth-brushing behaviour				
Twice daily or more	1.00 (ref.)		1.00 (ref.)	
Once daily	1.52 (0.99–2.34)	0.055	1.40 (0.88–2.22)	0.14
Less than once daily	3.99 (2.52–6.32)	<0.001	3.35 (2.02–5.57)	<0.001

*By simple regression analysis.

**Adjusted OR, goodness of fit by Hosmer and Lemeshow test (P = 0.997).
